# Supplementary material for: Association between oral microbiome and seven types of cancers in East Asian population: a two-sample Mendelian randomization analysis
Source: Front Mol Biosci. 2023 Nov 21;10:1327893. doi: 10.3389/fmolb.2023.1327893 (PMC10702768; doi:10.3389/fmolb.2023.1327893)
Supplement: Supplementary file 1 [file DataSheet1.ZIP › Supplementary Materials/MR plots for tongue/tongue═╝/Lung cancer/pheno.2550_to_lung cancer_forest.pdf]

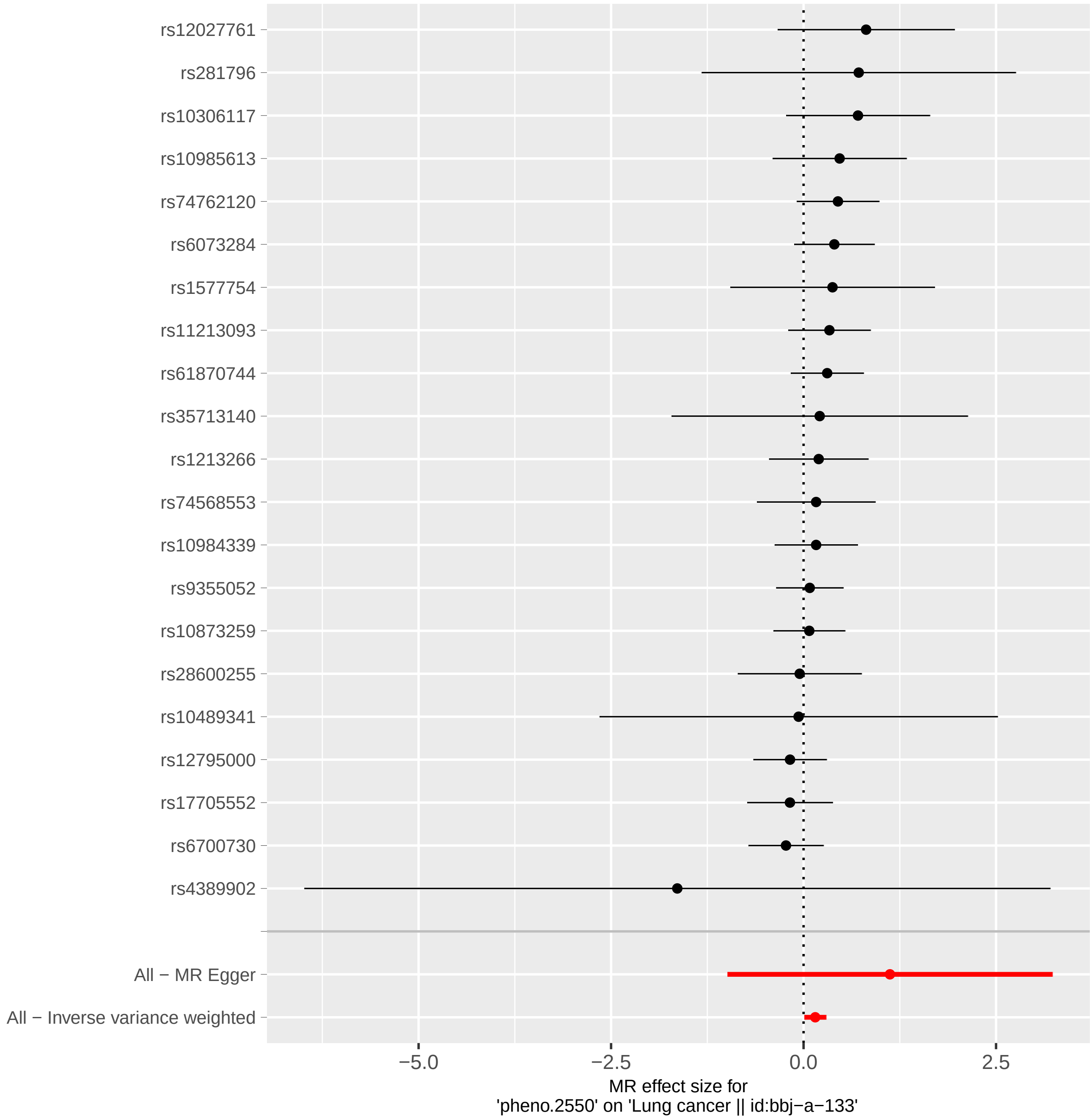

rs12027761
rs281796
rs10306117
rs10985613
rs74762120
rs6073284
rs1577754
rs11213093
rs61870744
rs35713140
rs1213266
rs74568553
rs10984339
rs9355052
rs10873259
rs28600255
rs10489341
rs12795000
rs17705552
rs6700730
rs4389902
All − MR Egger
All − Inverse variance weighted
−5.0
−2.5
0.0
2.5
MR effect size for
'pheno.2550' on 'Lung cancer || id:bbj−a−133'
